# Mapping a Decade of Physical Activity Interventions for Primary Prevention: A Protocol for a Scoping Review of Reviews

**DOI:** 10.2196/resprot.4240

**Published:** 2015-07-27

**Authors:** Leah Goertzen, Gayle Halas, Janet Rothney, Annette SH Schultz, Pamela Wener, Jennifer E Enns, Alan Katz

**Affiliations:** ^1^ Faculty of Kinesiology and Recreation Management University of Manitoba Winnipeg, MB Canada; ^2^ Department of Family Medicine, College of Medicine, Faculty of Health Sciences University of Manitoba Winnipeg, MB Canada; ^3^ Neil John Maclean Health Sciences Library University of Manitoba Winnipeg, MB Canada; ^4^ College of Nursing, Faculty of Health Sciences University of Manitoba Winnipeg, MB Canada; ^5^ Department of Occupational Therapy, College of Rehabilitation Sciences, Faculty of Health Sciences University of Manitoba Winnipeg, MB Canada; ^6^ Manitoba Centre for Health Policy Department of Community Health Sciences, College of Medicine, Faculty of Health Sciences University of Manitoba Winnipeg, MB Canada

**Keywords:** physical activity, primary prevention, protocol, scoping review

## Abstract

**Background:**

Physical activity is a key behavioral component for the primary prevention of noncommunicable disease. The uptake of physical activity is influenced by individual and broader factors including social, economic, and environmental conditions.

**Objective:**

The purpose of this paper is to describe a protocol for a scoping review of reviews (SRR) that aims to map a decade of research focused on physical activity interventions within the domain of primary prevention.

**Methods:**

The 5 stages of our SRRs design were adapted from a seminal scoping review methodology. Our search strategy was developed for the following databases: SPORTDiscus, PubMed, Scopus, the Cochrane Library, the Cumulative Index to Nursing and Allied Health Literature, PsycINFO, and Educational Resources Information Centre. Two reviewers (LG and AK) independently screened eligible studies and compared results to determine the final study selection. One reviewer will conduct the data extraction (LG); a second reviewer (AK) will assess the results to ensure comprehensiveness and accuracy of the scoping review synthesis.

**Results:**

The SRRs will provide a broad overview of the physical activity research literature specific to primary prevention, and will describe key features of physical activity interventions. Potential gaps in the physical activity action areas will be identified, and thus, the results will inform future research directions.

**Conclusions:**

This paper describes an innovative approach for comprehensively mapping an important topic’s research trends in the last decade.

## Introduction

### Background

Physical activity provides health benefits that include stress reduction, improved functional ability, and a key means of energy expenditure that contributes to weight control [1]. Conversely, physical inactivity is the fourth leading risk factor contributing to noncommunicable diseases (eg, cardiovascular disease, diabetes, cancer, and chronic respiratory disease), accounting for an estimated 6% of deaths globally [1,2]. Despite the fact that physical activity is recognized as a key factor for the primary prevention of diseases [3], physical activity levels continue to decrease globally despite an extensive range of interventions. Several researchers attribute this phenomenon to the complexity of behavior change to support physical activity [4,5].

### Physical Activity and Exercise

Broadly, physical activity is defined as any bodily movement produced by skeletal muscles, which requires energy expenditure [6]. Exercise remains a key subcategory of physical activity, which focuses on achieving aspects of physical fitness through planned, structured, repetitive, and purposeful movements [7]. Concepts such as health-enhancing physical activity, active living, leisure-time physical activity, active transportation, and household physical activities have also been added to physical activity promotion to encourage greater participation in a variety of settings [8,9]. The type of physical activity that may produce the greatest health-risk reduction has not yet been determined [10,11]. Evolving concepts of physical activity have expanded the scope of physical activity interventions, presenting new challenges for primary prevention intervention research and surveillance.

Enhancing physical activity is a complex behavior change that is influenced by multiple factors [5,11]. Over the past decade, population-level approaches have sought to address the broader factors that influence behavior including social, economic, and physical environments; personal health practices; individual capacity and coping skills; and health services [12,13]. In 2007, the World Health Organization (WHO) published a guide for population-based approaches to increase physical activity as part of a global strategy to improve health outcomes [14]. Several key action areas were identified for increasing physical activity including policy, education, promotion, and creating supportive social and physical environments. These may be combined to produce multistrategy interventions. It was also suggested that population-based interventions should be combined with tailored interventions targeting specific population groups, such as people at risk for developing noncommunicable diseases. Vulnerable or marginalized populations that experience greater risk for noncommunicable diseases also tend to have the lowest levels of physical activity [15,16].

The proliferation of physical activity literature over the last decade addresses many relevant aspects: different types and intensities of exercise, sport, and leisure-time physical activity and their effects on health; the influence of settings (barriers/facilitators) on physical activity behaviors; and policies developed in response to the alarming global trend of decreasing physical activity levels. Given the recognition of physical activity as important for preventing chronic disease, and the vast amount and complexity of published literature on this topic, a broad overview that maps physical activity research literature specific to primary prevention is warranted.

Reviews conducted in health disciplines tend to be systematic reviews of particular interventions or outcomes, but despite the rigorous results they produce to address specific questions, these methods are less useful when the aim is to address complex practice issues or broad research questions [17,18]. Scoping reviews have become increasingly used in response to a growing demand for effective and timely summaries of the breadth of primary research around a particular topic [19,20]. Here, we describe a protocol for a scoping review of reviews (SRR) to summarize the decade of primary prevention-focused physical activity interventions since the release of the WHO’s guidelines [14].

## Methods

### Scoping Review

A scoping review is an ideal methodology for mapping key concepts within a research area and for identifying main sources and types of evidence available when the research literature is vast or diverse, or both [21,22]. Scoping reviews are different from systematic reviews, which attempt to answer a specific research question by collating all empirical evidence that fits prespecified eligibility criteria [23]. Thus, in this SRR there will be no attempt to “weight” the evidence to answer a specific question. Rather, our goal is to map intervention trends and concepts, and to summarize these findings to identify potential gaps in research.

Our SRR approach is adapted from Arksey and O’Malley’s scoping methodology that describes up to 6 stages of the scoping review process [21]. An integral aspect of this methodology is a rigorous and transparent approach in each stage of the study design. We will adopt such an approach using an iterative process during each stage of the review to allow us to modify methods and record the methodological differences in an SRR compared with a scoping review of primary literature.

### Stage 1: Establishing the Research Questions

Similar to other review designs [21], the initial research questions shape the design of the SRR (Textbox 1). These questions were established using an iterative process that involved team discussions as we became more familiar with the literature. Several key research questions were derived from the WHO’s suggestions for population-based approaches for increasing physical activity [14]. We used the Preferred Reporting Items for Systematic Reviews and Meta-Analyses (PRISMA)-Equity 2012 Extension [24] to determine the equity-focused question and the operational definition. Because our SRR does not appraise the findings of the included reviews, we did not attempt to establish whether interventions are effective. Instead, we will indicate in a separate category those reviews that explicitly address factors contributing to intervention effectiveness or efficacy in their research aim, which may provide direction for future research.

**Overarching research questions.**

Which physical activity strategies are being addressed in the literature [14]?

- Individual-targeted interventions (eg, individual behavioral interventions)
- Education or promotion (national, regional, or local informational education or promotion)
- Supportive social environments (eg, counselors, mentors, role models)
- Supportive physical environments (relevant settings and opportunities that determine availability)
- Policy (government or organizational policy)
- Multicomponent interventions (ie, several health-related strategies in a single intervention)

Which individuals or groups are targeted in the physical activity literature [14]?

- Individuals (eg, children, youth, adults)
- Family
- Community
- Subpopulation (eg, age group)
- Sectors (eg, schools, workplaces, primary care providers)
- Society (ie, general population)

How is equity addressed in the physical activity review literature?

- Equity is explicitly stated in the research objective
- Includes equity categories based on Preferred Reporting Items for Systematic Reviews and Meta-Analyses-Equity 2012 Extension [24] such as gender, age, ethnicity

What factors are being researched that may influence physical activity uptake?

- Social and health determinants
- Correlates
- Mediators/Moderators
- Barriers

What are the trends in physical activity concepts, action areas, and population targets?

Which reviews explicitly examine intervention effectiveness or efficacy?

### Stage 2: Identifying Relevant Studies

Our SRR aimed to be comprehensive in identifying relevant studies, yet we limited our scope to include only published review literature to manage the vast quantity of physical activity literature. Team discussions established the eligibility criteria in the preliminary planning of the SRR analysis (Textbox 2). Similar to other scoping review methods [21], these criteria may be refined in later stages of the SRR.

A comprehensive search was performed in the following electronic databases: SPORTDiscus, PubMed, Scopus, the Cochrane Library, Cumulative Index to Nursing and Allied Health Literature, and Educational Resources Information Centre. Boolean terms “AND” and “OR” were used to build the keyword searches in the databases. We developed our search around physical activity intervention concepts and keywords that are broad, yet relate specifically to physical activity interventions within the domain of primary prevention (Multimedia Appendix 1). Our team librarian (JR) led the refinement of our database search strategies during this stage. Each search result was documented and the references were imported into separate folders using RefWorks reference management software.

**Eligibility criteria for the scoping review of reviews.**

- Inclusion criteria
  - Published in English
  - Human subjects
  - Date range January 2003 to June 2014
  - All age groups
  - Research that targets the general population where no illness/condition is identified.
  - Review methods specifically described a systematic review, meta-analysis, meta-synthesis, scoping review, rapid review, critical review, or described a systematic approach.
  - Research located in developed Westernized countries (Canada, United States, Europe, United Kingdom, Australia, and New Zealand).

- Exclusion criteria
  - Journal articles that are not rigorous reviews (ie, those not listed in the inclusion list), such as book reviews, opinion articles, commentaries, or editorial reviews.
  - Research targeting a population because of a diagnosed illness or disease or interventions targeting treatment of a specific disease, illness, or condition.
  - Research about direct health benefits from physical activity.
  - Research that focuses on research design (eg, methods, protocols, theories).

### Stage 3: Study Selection

We used a 2-stage study-selection process. In the first stage, a single reviewer applied the defined inclusion criteria (ie, interventions targeting healthy populations in developed countries) to titles and abstracts. Reviews that were overtly ineligible were removed, such as physical activity interventions that treated a previously existing health condition. All potentially eligible studies were then distributed to 2 independent reviewers (LG and AK) on the team. Each reviewer applied the eligibility criteria (Textbox 2) and any eligibility discrepancies were discussed between reviewers until consensus was reached or brought to the larger team for further discussion. For example, there was a discrepancy regarding physical activity interventions that targeted obesity as a health condition versus obesity as a risk factor. The team refined the eligibility criteria to include reviews that targeted obesity if the outcomes measured change in physical activity levels along with obesity-related outcomes.

### Stage 4: Charting the Data

The data-extraction tool was developed using an iterative team process. The preliminary data-extraction categories were derived from our overarching research questions (Textbox 1). As suggested by Daudt et al [20], each team member piloted the data-extraction tool independently, and the results were discussed as a team. At this stage, we used abstracts rather than full text to extract data and complete the chart. We determined that abstracts were a suitable source for data extraction based on the results of the pilot-extraction exercises.

Our data-extraction categories (Table 1) were derived from Arksey and O’Malley’s scoping review protocol [21], the WHO framework for increasing physical activity [14], and the PRISMA-Equity 2012 Extension [24]. Adjustments to the data-extraction tool were accomplished using team discussion and consensus. For example, we attempted to extract in-depth details about the effectiveness of intervention outcomes in the pilot phase of extraction. Following a team discussion, we determined we did not aim to establish whether interventions were effective. Rather, we wanted to map and narratively describe review questions that focused on intervention effectiveness or efficacy. Two reviewers (LG and AK) will independently extract the data and compare results. Discrepancies will be discussed between reviewers until consensus is reached or brought to the larger team for further discussion.

**Table 1:** Data-extraction tool.

Data	Details extracted
Article summary	Author
	Title
	Publication year
	Number of studies
	Date range
	Review type
Population	Age (eg, adults)
	Descriptors (eg, workplace)
Action areas	Policy
	Education/Promotion
	Supportive physical environments
	Supportive social environments
	Multicomponent interventions
Physical activity concept	For example, leisure-time physical activity
Intervention	Descriptors
	Objectives
	Measures
Review focus	Process
	Impact
	Outcomes
Equity	Explicitly stated? Yes/No
Equity-related categories	For example, ethnicity
Effectiveness/efficacy	Factors that contribute to intervention effectiveness or efficacy

### Stage 5: Collating, Summarizing, and Reporting the Results

This physical activity SRR will provide an overview of the breadth of physical activity research to inform our primary prevention research agenda. We will use Arksey and O’Malley’s methods of reporting and provide a descriptive analysis of the extent, nature, and distribution of the studies included in the review [21] as well as a narrative, thematic summary of the data collected. Our primary objective is to describe key categories such as target populations, dominant action areas, and intervention characteristics. We will discuss the types of questions posed in review research about intervention effectiveness and provide suggestions for future research. Potential gaps in the physical activity action areas will be identified based on our summary of the review literature.

## Discussion

### Preliminary Findings

Our study is a scoping review of published reviews that is not limited to systematic reviews. We chose a broader selection of review literature to comprehensively explore physical activity interventions aimed at primary prevention. Extracting data from a variety of reviews may prove difficult, because the included studies will have a wide range of methodological approaches, settings, study populations, and behaviors. However, our goal is to map trends rather than answer a specific question, which will provide novel insights with regard to future research needs to enhance current primary prevention policies and programs.

### Limitation

A potential limitation of this study may be a lack of quality assessment of the included articles, yet this is typical of a scoping review [18,20]. The use of abstracts may restrict our ability to provide conclusive knowledge claims about findings in the research field. Arksey and O’Malley [21] point out that scoping reviews are often conducted to inform future research. Thus, if we find there are limitations in the data from the abstracts, we have the opportunity to develop research questions related to a more specific topic and further explore a subset of the reviews.

### Conclusion

Physical activity is an important intervention for the primary prevention of noncommunicable diseases and the promotion of health. Research suggests interventions require a multidimensional approach that encompasses the broader social, economic, and environmental factors that influence behavior [13,16]. Our protocol for SRRs is an innovative approach for synthesizing comprehensive intervention research that provides an overview of the extent, range, and nature of physical activity research within the last decade. We have described the 5 stages underpinning our SRR protocol and we anticipate some iterative revisions based on the nature of scoping reviews. We are confident that our multicomponent data-extraction tool will provide new direction for physical activity interventions within the domain of primary prevention.

## Appendix

Multimedia Appendix 1 Search strategy for SPORTDiscus.

## Abbreviations

PRISMA: Preferred Reporting Items for Systematic Reviews and Meta-Analyses
SRR: scoping review of reviews
WHO: World Health Organization
