# Supplementary material for: Mapping a Decade of Physical Activity Interventions for Primary Prevention: A Protocol for a Scoping Review of Reviews
Source: JMIR Res Protoc. 2015 Jul 27;4(3):e91. doi: 10.2196/resprot.4240 (PMC4705362; doi:10.2196/resprot.4240)
Supplement: Multimedia Appendix 1 [file resprot_v4i3e91_app1.pdf]

Complete Search Results				
Database: Sport Discus				
#	**Query**	**Limiters/Expanders**	**Results**	**Eligible: Second Scan**
S25	S19 AND S16	Limiters - Published Date: 20030101-20131231; Language: English		
		Expanders - Apply related words		
		Search modes - Find all my search terms	2	1
S24	S19 AND S14	Limiters - Published Date: 20030101-20131231; Language: English		
		Expanders - Apply related words		
		Search modes - Find all my search terms	0	
S23	S19 AND S12	Limiters - Published Date: 20030101-20131231; Language: English		
		Expanders - Apply related words		
		Search modes - Find all my search terms	14	7
S22	S19 AND S10	Limiters - Published Date: 20030101-20131231; Language: English		
		Expanders - Apply related words		
		Search modes - Find all my search terms	25	8
S21	S19 AND S8	Limiters - Published Date: 20030101-20131231; Language: English		
		Expanders - Apply related words		
		Search modes - Find all my search terms	0	
S20	S19 AND S8	Limiters - Published Date: 20030101-20131231; Language: English		
		Expanders - Apply related words		
		Search modes - Find all my search terms	0	
S19	S18 AND S5	Limiters - Published Date: 20030101-20131231; Language: English		
		Expanders - Apply related words		
		Search modes - Find all my search terms	1,126	
S18	S1 AND S3	Limiters - Published Date: 20030101-20131231; Language: English		
		Expanders - Apply related words		
		Search modes - Find all my search terms	3,698	
S17	S7 AND S16	Limiters - Published Date: 20030101-20131231; Language: English		
		Expanders - Apply related words		
		Search modes - Find all my search terms	12	2
S16	"critical review"	Limiters - Published Date: 20030101-20131231; Language: English	246	

		Expanders - Apply related words<br>Search modes - Find all my search terms		
S15	S7 AND S14	Limiters - Published Date: 20030101-20131231; Language: English<br>Expanders - Apply related words<br>Search modes - Find all my search terms	3	2
S14	"meta synthesis"	Limiters - Published Date: 20030101-20131231; Language: English<br>Expanders - Apply related words<br>Search modes - Find all my search terms	20	
S13	S7 AND S12	Limiters - Published Date: 20030101-20131231; Language: English<br>Expanders - Apply related words<br>Search modes - Find all my search terms	123	17
S12	"meta analysis"	Limiters - Published Date: 20030101-20131231; Language: English<br>Expanders - Apply related words<br>Search modes - Find all my search terms	1,780	
S11	S7 AND S10	Limiters - Published Date: 20030101-20131231; Language: English<br>Expanders - Apply related words<br>Search modes - Find all my search terms	231	50
S10	"systematic review"	Limiters - Published Date: 20030101-20131231; Language: English<br>Expanders - Apply related words<br>Search modes - Find all my search terms	2,301	
S9	S8 AND S7	Limiters - Published Date: 20030101-20131231; Language: English<br>Expanders - Apply related words<br>Search modes - Find all my search terms	1	0
S8	("scoping review" OR "scoping study" OR "rapid review")	Limiters - Published Date: 20030101-20131231; Language: English<br>Expanders - Apply related words<br>Search modes - Find all my search terms	17	
S7	S6 AND S5	Limiters - Published Date: 20030101-20131231; Language: English<br>Expanders - Apply related words<br>Search modes - Find all my search terms	8,784	
S6	S1 AND S2	Limiters - Published Date: 20030101-20131231; Language: English<br>Expanders - Apply related words<br>Search modes - Find all my search terms	28,906	
S5	(efficacy OR effective OR evaluation OR	Limiters - Published Date: 20030101-20131231; Language: English<br>Expanders - Apply related words	140,256	

	assessment OR success* OR "best practices" OR	Search modes - Find all my search terms		
S4	(efficacy OR effective OR evaluation OR assessment OR success* OR best practices" OR "promising practices")	Limiters - Published Date: 20030101-20131231; Language: English	0	
		Expanders - Apply related words		
		Search modes - Find all my search terms		
S3	(barrier* OR determinant* OR polic* OR advoca*)	Limiters - Published Date: 20030101-20131231; Language: English	29,360	
		Expanders - Apply related words		
		Search modes - Find all my search terms		
S2	(intervention OR prevention OR program* OR promot* OR strateg*)	Limiters - Published Date: 20030101-20131231; Language: English	140,367	
		Expanders - Apply related words		
		Search modes - Find all my search terms		
S1	("physical activity" OR exercise)	Limiters - Published Date: 20030101-20131231; Language: English	92,739	
		Expanders - Apply related words		
		Search modes - Find all my search terms		
			TOTAL	87
